# Optical Biosensors for Blood Coagulation Monitoring: Advantages, Limitations, and Translational Potential

**DOI:** 10.3390/bios16020123

**Published:** 2026-02-16

**Authors:** Zichen Wang, Gaohong Di, Jing Wang

**Affiliations:** 1 Department of Biomedical Engineering, College of Life Science and Technology, Huazhong University of Science and Technology, Wuhan 430074, China; zichenwang@student.unimelb.edu.au; 2 Faculty of Science, University of Melbourne, Parkville, VIC 3010, Australia; 3 Department of Anesthesiology, Union Hospital, Tongji Medical College, Huazhong University of Science and Technology, Wuhan 430022, China; gdi@ucm.es; 4 Departamento de Farmacología y Toxicología, Facultad de Medicina, Universidad Complutense de Madrid, 28040 Madrid, Spain

**Keywords:** optical biosensors, coagulation monitoring, hemostatic assessment, clinical application, point-of-care testing

## Abstract

Dynamic monitoring of hemostatic equilibrium is indispensable for clinical safety in high-risk scenarios, while current clinical methods are limited by sample volume, detection speed, and physiological relevance. These shortcomings underscore the demand for novel sensing platforms. Optical biosensors, leveraging label-free detection, rapid response, and multi-level characterization, could serve as a transformative solution for decentralized and point-of-care monitoring. This review systematically summarizes advances in optical coagulation testing, encompassing light transmission aggregometry, laser speckle rheology, optical coherence tomography/elastography, optic–acoustic coupled methods, and fluorescence biosensing. These technologies complementarily capture structural and mechanical and some molecular and cellular dynamics of coagulation, bridging gaps in traditional assays. Despite promising preclinical and clinical correlations, translation barriers persist in lack of standardization of metrics, interference mitigation, and multi-center validation in diverse patient cohorts. Future development of optical biosensing platforms for coagulation testing should focus on modular integration, AI-aided interference correction, and microfluidic miniaturization to realize actionable, real-time coagulation assessment. Optical biosensors hold unparalleled potential to transform hemostatic monitoring from static endpoint testing to dynamic, interpretable evaluation, guiding personalized clinical decisions.

## 1. Introduction

Hemostasis is the core defense mechanism for the body to maintain vascular integrity and circulatory stability. Hemostasis involves multiple biological, chemical, and physical processes [1,2] such as coagulation factor cascade reactions, platelet adhesion and aggregation, and the formation and degradation of fibrin networks. More specifically, this process spans multiple levels, including molecular, cellular, microstructural, and global mechanical aspects [1,3]. Enzymatic reactions and their regulatory networks at the molecular level govern the initiation, amplification, and inhibition of the coagulation factor cascade; platelet adhesion, activation, and aggregation provide mechanical support and reaction platforms for the formation of initial hemostatic plugs, crucially affecting the formation efficiency and functional stability of the plugs; the generation and continuous remodeling of fibrin networks shape the spatial structural characteristics of clots, significantly affecting their structural integrity and shear resistance; the temporal evolution of the global viscoelasticity of clots and the subsequent fibrinolysis process greatly influence the success of hemostasis and the balance between thrombosis and thrombolysis. Meanwhile, this process is an exquisite dynamic equilibrium maintained by the mutual checks and balances of procoagulant, anticoagulant, and fibrinolytic mechanisms. For instance, after tissue injury, the coagulation system needs to be rapidly activated to control bleeding while also avoiding excessive activation that may trigger pathological thrombosis. Therefore, rather than a static judgment at a single time point, the assessment of coagulation status should ideally be a continuous monitoring of this dynamic equilibrium process. In high-risk scenarios such as organ transplantation [4,5], anticoagulant therapy [6], cardiac surgery [7], and Extracorporeal Membrane Oxygenation (ECMO), this balance could quickly deviate significantly. Thus, comprehensive, rapid, continuous, and accurate assessment of the dynamic coagulation equilibrium is of paramount importance for guiding clinical decisions and ensuring patient safety.

However, current conventional clinical coagulation testing systems, including stepwise screening tests and viscoelastic testing (VET) [8], are yet to meet the need of comprehensive, rapid, continuous, and accurate multi-scale assessment of dynamic coagulation equilibrium. Stepwise screening tests are based on the theoretical framework of “primary/secondary hemostasis—fibrinolysis” and localize potential pathway defects through a series of sequential experiments such as Prothrombin Time (PT), activated Partial Thromboplastin Time (aPTT), fibrinogen (FIB) determination, Thrombin Time (TT), D-dimer, and platelet count [1,9,10]. Although such methods have mature reference standards under laboratory conditions, their outputs are mostly endpoint times or steady-state indicators. Hence, these tests struggle to provide direct information on the continuous evolution of kinetic, structural, and mechanical properties during coagulation. Notably, these assays are largely plasma-based and performed under highly standardized (and non-physiological) reagent conditions. Thus, they primarily report pathway-specific clotting endpoints rather than whole-blood clot mechanics, spatial heterogeneity, or shear-dependent hemostasis. In addition, their long turnaround time and complex testing processes greatly limit their applications in scenarios requiring rapid decisions such as intraoperative and point-of-care (POC) settings. VET technologies like Thromboelastography (TEG) and Rotational Thromboelastometry (ROTEM), having strong resistance to matrix interference, reflect “processual” information, including coagulation initiation, clot growth, clot strength, and fibrinolysis, to a certain extent by recording the entire viscoelasticity curve of whole-blood transformation from fluid to clot. However, because VETs mainly provide global, macroscale viscoelasticity curves, they offer insufficient direct characterization of clot microstructure formation, local heterogeneity, and molecular mechanisms of the target reaction, limiting their ability to distinguish between different pathophysiological mechanisms. For example, similarly low maximum amplitude (MA) values may result from platelet dysfunction or fibrinogen deficiency, which are difficult to be reliably identified by a single global VET curve alone. Furthermore, the measurement principle of VETs does not recapitulate vascular shear environments and does not directly report molecular/cellular activation states; consequently, mechanistic attribution and shear-dependent hemostasis remain challenging using only VETs [11,12,13,14].

Therefore, to achieve continuous monitoring of the hemostatic dynamic equilibrium process across molecular, cellular, structural, and mechanical levels, biosensing technologies with diverse detection mechanisms and facile multimodal combination are particularly critical. Optical biosensing has demonstrated unique advantages in response to this demand: the interaction between light and biological systems inherently relies on and exhibits diverse physical mechanisms, enabling natural correspondence to information sources at different levels of the coagulation process. This methodological diversity enables optical biosensing to complementarily characterize multiple aspects of the coagulation process—from spatial organization of fibrin networks to the kinetic features of the temporal evolution of global viscoelasticity, and some to specific reactions and functional states at the molecular and cellular levels.

**Figure 1:**
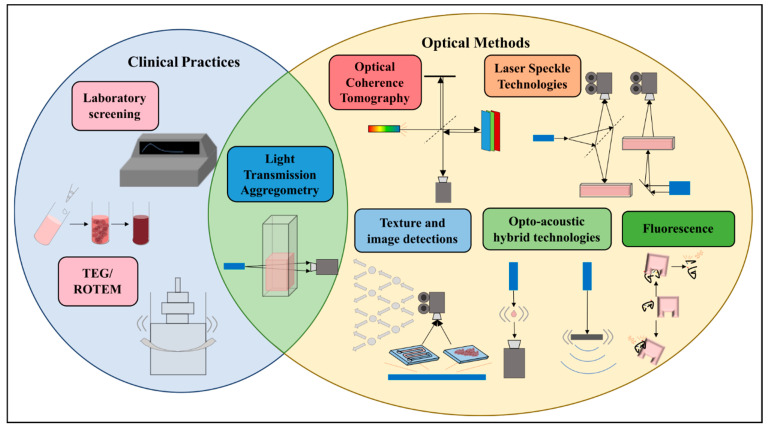
Optical biosensors for monitoring dynamic coagulation process. Clinical practices include viscoelastic tests (TEG/ROTEM) and laboratory screening assays. Optical-based methodologies comprise light transmission aggregometry (LTAs), laser speckle (LS) technologies, image texture-based detection, optical coherence tomography (OCT), opto-acoustic technologies, and fluorescence assays; their respective measured parameters are also depicted in the diagram.

In specific coagulation testing practices, this methodological diversity has developed into a series of technical approaches targeting different coagulation levels and application scenarios. As a classic reference, light transmission aggregometry (LTA) evaluates platelet aggregation through changes in transmitted light intensity and still occupies an important position in platelet function research and laboratory testing. In terms of rapid characterization of the global coagulation process, biosensing technologies based on transmitted/reflected light intensity and image texture can capture the overall process of blood transformation from fluid to gel with a low technical threshold. In terms of micro-rheological kinetic characterization, laser speckle (LS) and related optical correlation analysis methods utilize the spatiotemporal decorrelation behavior of speckle patterns and are highly sensitive to initial changes such as viscoelasticity enhancement during coagulation; this makes them particularly suitable for rapid detection of small samples. In terms of characterizing the microstructure and mechanical properties of clots during coagulation, optical coherence tomography (OCT) can non-invasively provide high-resolution three-dimensional structural characterization of fibrin networks and clot formation; fluctuations in its phase and intensity can also reflect certain mechanics-related information; while the mechanical extension technology of OCT, optical coherence elastography (OCE), could quantify viscoelasticity evolution. Multimodal platforms combining optics and acoustics enhance the ability to detect deep tissue microclots or structural changes by introducing acoustic contrast mechanisms. At a molecular level, fluorescence sensing technology can directly report the activity of specific coagulation factors, fibrin generation, or drug action targets, providing higher specificity for coagulation mechanism analysis and pathway abnormality interpretation (as summarized in Figure 1).

## 2. Light Transmission Aggregometry

In the multi-level dynamic process of coagulation, platelet function is usually regarded as a key link that is activated earliest in the hemostatic response [3]. Platelet adhesion, activation, and aggregation not only directly affect the formation efficiency of initial hemostatic plugs but also provide important reaction interfaces for subsequent coagulation factor cascade reactions and fibrin network construction. Therefore, detection methods established around platelet aggregation behavior have long constituted an important part of the coagulation function evaluation system, and they are relatively independent in clinical practice.

**Figure 2:**
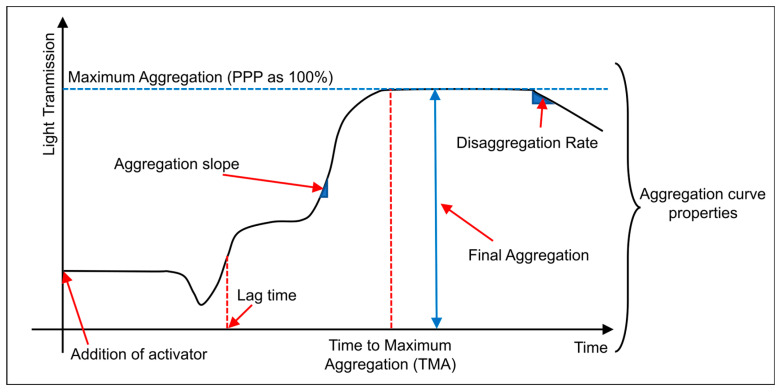
Typical LTA traces with key parameters annotated. Aggregation intensity and stability parameters include maximum aggregation, final aggregation, and disaggregation rate. Aggregation kinetic parameters include time to maximum aggregation (TMA), lag time, and aggregation slope. Intensity and stability parameters quantify the magnitude and sustainability of platelet aggregation—a metric critical for evaluating whether platelet function is sufficient to prevent bleeding or prone to excessive thrombosis—whereas kinetic parameters characterize the temporal dynamics of platelet activation and aggregation, which provides key insights into the speed and progression of platelet responses to guide clinical assessment of hemostatic competence. Blue triangles denote the aggregation slope.

Among the numerous detection methods, light transmission aggregometry (LTA) is one of the earliest optical methods systematically introduced for platelet function evaluation, and it is still widely used as a classic reference method for laboratory testing and mechanism research [15,16,17,18]. Its basic principle is as follows: upon addition of specific agonists, such as ADP, epinephrine, collagen, arachidonic acid, ristocetin, etc., to platelet-rich plasma (PRP), platelet activation and mutual aggregation leads to clump formation, thereby reducing sample turbidity and increasing sample light transmittance. Instruments usually obtain aggregation curves by continuously recording changes in transmitted light intensity over time, using platelet-poor plasma (PPP) as the 100% light transmission baseline and PRP as the 0% light transmission starting point. From the curves obtained, key indicators such as the maximum amplitude of platelet aggregation, aggregation rate (slope), initiation time, and aggregation persistence can be extracted (Figure 2). On this basis, combining different agonists and inhibitor conditions, LTA can provide functional evaluation of a specific receptor or signal pathway, which is used for auxiliary diagnosis of congenital or acquired platelet dysfunction, monitoring of antiplatelet therapy response, etc.

LTA has strict requirements for sample preparation and operation procedures, as they need strict centrifugation conditions, target range of platelet count in PRP, proper source and preservation of agonists, rigorous sample addition sequence and mixing method, temperature control and stirring conditions, etc. These parameters significantly affect the curve shape and threshold interpretation of LTA. Over the past ten years, LTA has made significant progress in automation and high-throughput screening, standardizing parameter adjustments, reducing operation threshold, improving throughput, and enhancing the reproducibility of results [18]. Microplate aggregation tests based on 96-/384-well plates [15,19] can simultaneously complete multi-agonist combinations or multi-sample screening in one plate, improving throughput and facilitating drug response spectrum analysis. Some studies have combined transmission aggregation reading with microfluidic systems [20] to reduce sample volume and shorten the detection process, providing new possibilities for applications in POC and resource-constrained environments.

Derived and extended techniques based on light transmission aggregometry (LTA) have undergone rapid development and been enriched. For example, luminescent aggregometry [18] combines traditional LTA with fluorescence or chemiluminescence probes to synchronously detect platelet aggregation and secretion functions like ATP release, enabling multi-dimensional characterization of platelet activation processes. Some studies have used LTA as a benchmark method for new optical biosensing technologies employing flow cytometry [21] or microscopic imaging [22]. In this sense, LTA is both a reference standard for platelet function evaluation in clinical practice, and an important control system for the design and performance verification of next-generation optical coagulation biosensors.

Nevertheless, LTA still has unavoidable limitations. Firstly, LTA suffers difficulties in direct extrapolation from its result to the hemostasis/thrombosis process under whole-blood and in vivo blood flow environments because it lacks physiological shear stress system and it uses PRP as the reaction system, lacking physiological elements such as red blood cells (RBCs), white blood cells, and plasma protein networks. Secondly, the sensitivity to preprocessing and operation of LTA has long posed challenges to cross-laboratory standardization and consistency of result interpretation. Thirdly, the sample preparation and detection processes determine that LTA is more suitable for central laboratories, while POC applications require rapid turnaround and pediatric/low-blood-volume populations. These limitations have prompted researchers to develop more miniaturized, integrated optical platforms that are closer to physiological conditions: retaining the sensitivity of optical signals to platelet function, researchers are gradually introducing strategies such as whole-blood measurement and microfluidic shear environments.

## 3. Texture-/Image-Driven Macroscopic Coagulation Characterization

**Figure 3:**
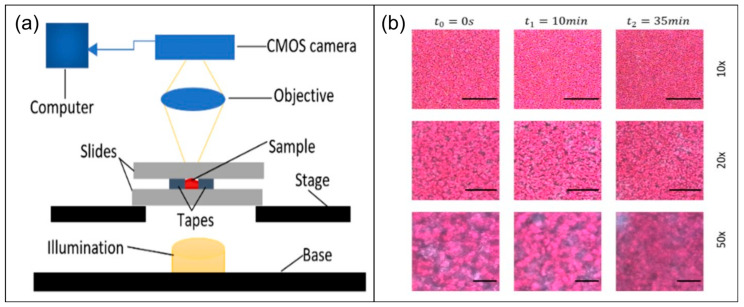
Apparatus and illustrations for coagulation tests based on texture detection and image processing. (**a**) Example of common texture-based optical coagulation biosensors. Figure reproduced from ref. [23]. CC BY License, MDPI, Copyright © 2021. (**b**) Example of data acquisition; the displayed graph shows continuously captured clotting images processed to visualize optical intensities. Figure reproduced from ref. [23]. CC BY License, MDPI, Copyright © 2021.

Unlike LTA mainly evaluating platelet aggregation in PRP systems, texture-/image-driven optical coagulation sensing methods enable macroscopic characterization of the coagulation process by tracking temporal changes in texture images of whole-blood samples over time; therefore, they offer greater potential for rapid assessment of the “overall coagulation function”. The core principle of texture-/image-driven methods is to translate the combined effects of key coagulation elements, like RBCs, platelets, and fibrin network remodeling, into measurable optical changes. These optical changes encompass light intensity, grayscale distribution, and spatial texture, from which quantifiable spatiotemporal features are extracted. This approach enables macroscopic characterization of coagulation spatiotemporal kinetics using low-cost, low-complexity imaging systems (Figure 3a).

From a physiological and optical perspective, the progression of the coagulation process causes systematic changes in the spatial distribution of scattering particles inside the sample [1]. As fibrinogen gradually cross-links to form insoluble fibrin networks, RBCs and platelets aggregate, rearrange, and embed into the newly formed clot structure, leading to the corresponding optical changes in transmission, reflection, and scattering paths of light. These changes manifest with the continuous evolution of overall brightness, contrast, granularity, and texture structure on the imaging plane (Figure 3b). Therefore, the temporospatial changes of image texture can be regarded as the projection of structural and rheological evolution during coagulation in the macroscopic imaging domain.

Technically, texture-/image-driven methods usually track changes in average image brightness, total transmitted/reflected light intensity, gray variance, or contrast over time; they usually employ the time when the signal first crosses a threshold the inflection point as the operational indicator of coagulation initiation or endpoint. Compared with a single curve, imaging can directly observe or even quantify spatial heterogeneity that may exist during coagulation, such as local clot generation, growth front advancement, or local blockage in microfluidic channels. Recently, the emerging approach attempts to enable models to automatically learn features from continuous texture image sequences with ML, realizing the expansion from single time point determination to multi-index classification or stage recognition.

Recently, Xu et al. collected a time series of images of blood flow in channels in microfluidic chips and used the stop time of the flow as the macroscopic reading of coagulation time [24]. The detection time of the system could be shortened to the second range (approximately 25–84 s). Plus, in 47 clinical samples, the stop time obtained from their image analysis demonstrated a good correlation with the international normalized ratio (INR) measured by ACL-TOP 750 (R^2^ ≈ 0.90) [24]. Louka and Kaliviotis [23] performed whole-blood coagulation evaluation without using exogenous activators; they characterized the coagulation process through the average intensity and kinetic features of color channels. This work highlighted the feasibility of assessing macroscopic clotting progression while retaining native intrinsic coagulation dynamics, although the detection time is in the order of tens of minutes. Further, Chen et al. [25] developed an ML approach by integrating texture feature extraction with models composed of convolutional neural networks (CNNs) and fully connected layers. Based on continuous image sequences, this ML approach generates clear, interpretable classification results for key clinical indicators of coagulation factor activity, fibrinogen function, and composite status classification. Validated against physical diagnosis, this approach achieved high accuracy in prospective double-blind samples, highlighting its potential to extend towards “multi-index, automated interpretation”.

Despite the advantages in cost and accessibility, texture-based optical coagulation biosensing methods still face several common challenges in clinical translation. Macroscopic image features are susceptible to the complexity of samples [26] and external interference; for example, hemolysis, icterus, lipemia (HILs) [26], changes in hematocrit, and differences in illumination and imaging geometry can significantly change intensity and texture statistics, placing high demands on the robustness and standardization of the system. In addition, because these methods mainly perceive pre-existing structural remodeling, their sensitivity to early coagulation events such as initial fibrin formation or slight viscoelastic changes is limited. On the other hand, once ML models are introduced, the methodology would be highly dependent on large-scale, cross-center transferable datasets with unified annotation and verification systems; otherwise, it will be difficult to ensure generalizability due to limitations in anti-interference and early coagulation detection.

In general, texture-/image-driven optical coagulation biosensing technology is an important technical direction for decentralized detection and POC applications, achieving rapid, spatial characterization of the macroscopic coagulation process with considerably low system complexity. However, its inherent limitations in sensitivity to early events and mechanism resolution prompt researchers to further develop optical strategies that are more sensitive to the initial structural and microkinetic changes in coagulation to compensate for the shortcomings of texture methods.

## 4. Laser-Speckle-Based Optical Coagulation Biosensing

**Figure 4:**
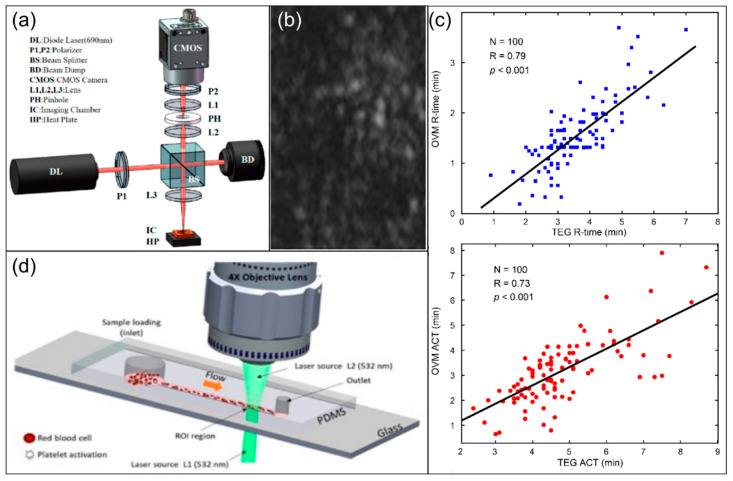
Fundamentals of laser speckle (LS)-based optical coagulation biosensing. (**a**) Common apparatus setup of LS-based optical coagulation biosensors. Figure reproduced from ref. [27]. CC BY license, MDPI, copyright © 2022. (**b**) Raw observation of optical coagulation biosensing signals using LS. Figure reproduced from ref. [28]. CC BY license, MDPI, copyright © 2024. (**c**) Correlation plot between reaction time (R-time) and activated clotting time (ACT) of TEG and coagulation time measured by optical vortex tracking in LS, with moderate correlations demonstrated. Figure reproduced from ref. [27]. CC BY license, MDPI, copyright © 2022. (**d**) Integration of LS optical coagulation biosensing with microfluidics for simulation to physiological shear system. Figure reproduced from ref. [28]. CC BY license, MDPI, copyright © 2024.

Compared to texture-/image-driven methods, laser speckle (LS) biosensing technology is more promising in providing quantitative evaluation of coagulation kinetics for early coagulation dynamics, as it focuses more on capturing micro-level information on the viscoelastic changes in whole-blood samples while maintaining a relatively simple optical path (Figure 4a). Its core principle relies on the interference of coherent light: when a coherent laser irradiates a blood sample, RBCs, platelets, and other endogenous particles produce multi-path scattering; such scattering generates scattered waves with different optical paths coherently superimposed on the detector plane and forms a random granular speckle pattern with bright and dark distributions (Figure 4b). In addition, under different coagulation phases and situations, the speckle behavior varies. While the scattering particles continuously undergo thermal motions inducing changes in their scattering phases, the speckles decorrelate rapidly over time; with the generation and cross-linking of fibrin networks and the advancement of gelation, the viscoelasticity of the system significantly increases and the movements of scattering particles are restricted, and the speckle decorrelation rate decreases accordingly. The LS method quantifies changes in speckle spatiotemporal statistics to map this slowing of micro-kinetics into quantifiable metrics of the coagulation process [1,2].

Among numerous LS-based implementations, laser speckle rheology (LSR) is one of the most widely used types. Starting from the statistical characteristics of temporal fluctuations in speckle intensity, LSR converts the temporal decorrelation behavior of speckles into a frequency-dependent viscoelastic modulus (G*(ω)). Through the real-time tracking of changes in G*(ω), LSR can plot a complete coagulation kinetic curve, from which key parameters highly corresponding to the clinical gold standard, TEG/ROTEM, can be extracted; these parameters include reaction initiation time (R), clot formation time (κ), alpha angle (clot growth rate), and MA (representing clot stiffness) [2,29]. A number of studies, represented by iCoagLab developed by Nadkarni [30,31], have made comparisons with results from conventional coagulation tests (CCTs) and clinical VETs [29]. These studies have demonstrated moderate correlation and consistency with reference methods, establishing LSR as an emerging optical viscoelasticity evaluation method with substantial room for further advancement.

Other LS methods directly realize detection through empirical correlations between speckle statistics and coagulation status without the need to fully reconstruct the viscoelastic spectrum: changes in pixel similarity, speckle contrast, or power spectrum characteristics between consecutive speckle frames can be used as operational readings for coagulation initiation or endpoint. Some studies established a direct high correlation between speckle kinetic characteristics and clinical indicators such as INR (Figure 4d) [32] and reaction initiation time of TEG [33,34] through regression or prediction models. Other studies have begun to explore more structured speckle analysis methods, such as tracking the movement of feature points in the speckle field to characterize the intensity of speckle fluctuations; preliminary studies suggest that the movement of zero-intensity points, a type of feature points, is correlated with coagulation initiation parameters, or activated clotting time (ACT), and other indicators (Figure 4c) [27]. Further studies have shown that the movement of feature points suggests changes in spatial heterogeneity during coagulation and that it could be modeled by the sub-diffusive motion, providing new possibilities for extending from traditional intensity statistics to higher-dimensional features and kinetic mechanism research. LS technology has also been extended to monitoring scenarios closer to physiological conditions or higher risks. Label-free evaluation of adhesion and aggregation kinetics could be performed while simulating physiological shear flow (Figure 4d) [28]. This leverages the combination of microfluidic technology and LS sensing; LS-derived speckle contrast, decorrelation time, or effective speckle size reflect particle motion status and the aggregation process. In cardiopulmonary bypass, a high-risk clinical scenario, Guzman-Sepulveda et al. [35,36,37] developed a compact LS sensing system to continuously monitor cardiopulmonary bypass. The LS system uses a spatiotemporal coherence gating strategy [35], taking the logarithmic slope of the power spectrum of the speckle intensity time series as a sensing indicator of coagulation capacity, and they proved its high correlation with results from traditional detection methods like TEG and CCT [36]. Preliminary tests of the system in approximately 40 cardiopulmonary bypass patient samples have demonstrated its potential in intraoperative real-time monitoring [36,37].

Despite the promising development prospects of LS biosensing technology, its clinical translation still faces several practical challenges. Firstly, the complexity of optical and physiological states, especially interfering factors, of the sample have not been systematically studied. Secondly, LS detection is sensitive to environmental conditions, including temperature fluctuations, ambient light, and mechanical vibrations. Currently, more comprehensive engineering and standardization solutions remain to be established for complex environments like operating rooms and Intensive Care Units (ICUs). Thirdly, the boundaries of methodological assumptions and parameters need to be further clarified, such as the influence of multiple scattering conditions, quantification of system parameters and empirical parameters, heterogeneous samples, and non-equilibrium processes on the speckle–mechanics mapping relationship. Finally, existing clinical validations are mostly single-centered and small-sampled, while systematic evaluations of populations with complex coagulation disorders and patients receiving direct oral anticoagulants (DOACs) are still insufficient. Establishing a more robust evidential framework and standardized clinical interpretation paradigm via multi-center, large-cohort studies is urgently required.

## 5. Optical Coherence Tomography/Elastography

While LS methods can sensitively capture kinetic information of the coagulation process, their signals lack depth resolution. To further obtain three-dimensional structural information of clot formation and its interactions with edges like microchannel walls/vascular walls, researchers introduced optical coherence tomography (OCT) based on low-coherence interference, as well as further realized active quantification of clot mechanical evolution on the basis of tomographic imaging through optical coherence elastography (OCE).

OCT itself is a depth-gated imaging technology based on low-coherence light interference, which can provide micron-level structural resolution within a depth range of millimeters [38]. OCT collects and reconstructs tomographic structures through coherent gating of backscattering at different depths. In coagulation detection, the value of OCT lies not only in static morphological imaging but also in its ability to extract dynamic statistics and structural evolution characteristics related to coagulation in a depth-resolved perspective. Mechanistically, scatterers in blood undergo continuous microscopic movement and rearrangement, causing temporal fluctuations in OCT intensity and phase signals. With the generation, cross-linking, and gelation of fibrin networks, the viscoelasticity of the system significantly increases and restricts the movement of scatterers, and the temporal fluctuations in OCT signals slow down accordingly. Therefore, by analyzing the temporal statistical characteristics of OCT signals, the kinetic evolution of the coagulation process can be indirectly reflected.

**Figure 5:**
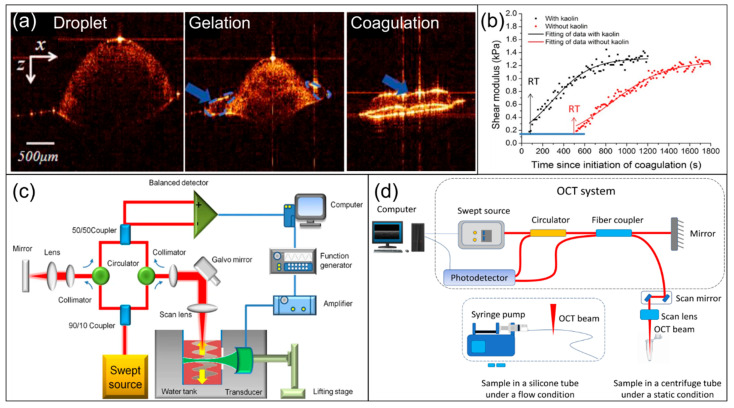
Illustrations and applications of optical coherence tomography (OCT) and optical coherence elastography (OCE). (**a**) An example of OCT-based coagulation monitoring: OCT images of a blood droplet in the droplet, gelation, and coagulation states, which are subsequently processed via machine-learning-based classification. Figure reproduced with permission from ref. [41], copyright © 2025, Wiley-VCH GmbH. (**b**) OCE-derived data for assessing coagulation properties following kaolin addition: reaction time—a parameter defined as the time from test initiation to the start of clot formation—is shown, with a significant difference in RT observed between assays with and without kaolin. Figure reproduced from ref. [42]. CC BY license, Springer Nature Limited, copyright © 2016, the authors. (**c**) Schematic presentation of experimental setup of OCE employing Doppler OCT. Figure reproduced from ref. [42]. CC BY license, Springer Nature Limited, copyright © 2016, the authors. (**d**) Schematic presentation of the system detecting blood coagulation dynamics based on OCT-SD. Figure reproduced with permission from ref. [39]. Copyright © 2025, Wiley-VCH GmbH.

To capture these kinetic changes in coagulation, studies often use time series of continuous depth acquisition at fixed lateral positions, or M-scans, to construct time–depth signal maps and calculate indicators like speckle standard deviation (Figure 5d) [39] or temporal autocorrelation function [40] within specific depths or regions. These studies indicated that with the progression of coagulation, the intensity fluctuation amplitude, or the speckle standard deviation, tends to decrease, while the autocorrelation decay time increases. On this basis, coagulation kinetic curves can be plotted and coagulation initiation or phasic turning points can be defined. In addition to analytical criteria based on statistical quantities, Li et al. used time series of B-scans of blood droplets (depth-lateral two-dimensional cross-sectional scanning, Figure 5a) combined with deep learning models for automatic recognition and quantification of coagulation stages [41]; this system classifies coagulation stages into liquid, gel, and coagulation through VGG net, a kind of CNN, and realizes pixel-level segmentation and conducts three-dimensional reconstruction of blood drop regions using U-Net, following the derivation of parameters including reaction time and clot growth rate from structural evolution.

Building on high-sensitivity detection of OCT, OCE introduces active mechanical excitations like mechanical vibration, piezoelectric excitation, or acoustic radiation force [43]. It leverages the sub-wavelength displacement resolution of phase-sensitive [44] or Doppler-based OCT (Figure 5c) [42] to track the resulting micro displacement field and maps the propagation of shear waves or elastic waves within the sample. OCE methods generally measure propagation speed, dispersion, and attenuation and build corresponding viscoelastic models and boundary condition assumptions for inversion. Following that, the evolution curve of shear modulus or equivalent viscoelastic parameters over time can be obtained, and quantitative tracking of the clot development process can be realized. Compared to LSR inferring viscoelasticity from speckle kinetics, OCE offers a distinct advantage in that it can provide mechanical parameters like shear modulus with standard physical units. This enables direct quantitative assessment of clot strength, stability, and the effects of interventions like drugs (Figure 5b) [42] and dilution [44] on clot mechanics, laying the foundation for comparability and mechanism interpretation of results across different conditions. Surrounding OCE, studies realized shear wave tracking in whole-blood or coagulation models and constructed mechanical indicators related to coagulation initiation, growth rate, and maximum strength.

Despite the promising prospects, the clinical translation of OCT/OCE still faces key limitations. For OCT, strong scattering of blood restricts the effective imaging depth to the millimeter level [43], limiting the imaging ability for deep thrombi or large-volume thrombi. Additionally, high system complexity, size, and cost, as well as enormous data and processing under continuous monitoring, pose higher requirements for engineering implementation. Furthermore, the OCT coagulation metrics defined in different studies, such as variance threshold, decorrelation time, and stage classification output, still lack unified standards, and their corresponding relationship, comparability, and multi-center validation against clinical gold standards remain to be further established. For OCE, the introduction of excitation sources increases cost and system complexity; variations in excitation frequencies, amplitudes, geometric boundaries, and data processing algorithms can significantly alter the inversion results, rendering standardization and cross-platform comparability of the excitation–acquisition–inversion pipeline a critical bottleneck. Meanwhile, spatial heterogeneity and time dependence of the coagulation system further complicate model selection and parameter quantification.

In summary, OCT/OCE provides a multi-level observation window encompassing structural, kinetic, and mechanical coagulation monitoring: OCT is good at visualizing the spatiotemporal structural evolution of thrombosis and extracting depth-resolved dynamic metrics, while OCE paves the way for direct quantification of clot mechanical properties. However, OCT and OCE for coagulation monitoring are still mainly in the exploration and early verification stage, and further advancements are required in multi-center clinical validation, metric standardization, system engineering, and detection time optimization. Notably, OCT is capable of dynamically tracking thrombosis formation and evolution in simulated physiological environments, i.e., vessels-on-chip [45]. This demonstrates the strength of in vivo monitoring of OCT, indicating its potential for visual and quantitative applications in traumatic hemorrhage management, anticoagulant efficacy monitoring, and novel antithrombotic drugs development.

## 6. Optic–Acoustic Coupling

Although OCT/OCE improves depth resolution, purely optical methods still face penetration limitations caused by strong tissue scattering and interface-related interference in some implementations. To this end, researchers have integrated acoustic modalities to construct an optic–acoustic synergy sensing strategy: by utilizing the deep penetration and mechanical effects of sound waves, stronger deep contrast can be obtained, and with the introduction of optical absorption information, structural and mechanical changes in deep tissues can be captured.

In specific implementations, a representative optic–acoustic synergy platform is centered on single-droplet rheological measurement coupled with acoustic excitation and optical reading. Kasireddy et al. proposed a non-contact single-droplet acoustic tweezer spectral biosensing technology [46]. This technology suspends whole-blood droplets of 4–6 μL in an acoustic field, induces controlled morphological vibration of the droplets through amplitude-modulated acoustic signals, and synchronously records temporal changes in droplet amplitude and morphology using light transmission measurements and camera imaging. By analyzing the droplet vibration mode and attenuation behavior, the equivalent viscosity and elastic modulus can be inferred. From the equivalent viscosity and elastic modulus, the evolution curve of viscoelasticity during the entire coagulation process, and hence the coagulation reaction time, can be obtained. While providing the reaction time with strong correlation with a standard coagulation screening indicator, PT/aPTT, this system additionally provides mechanical readings related to clot strength or stability, enabling the joint output of temporal and mechanical indicators with an extremely small sample volume.

**Figure 6:**
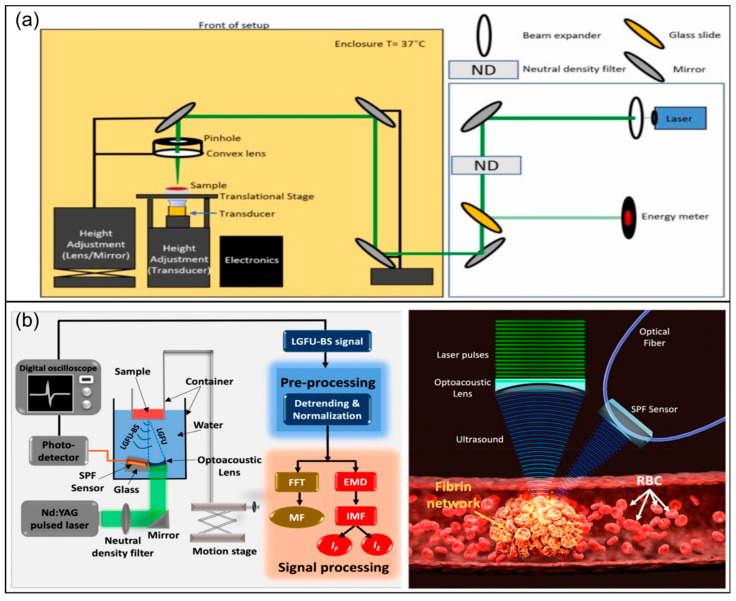
Typical mechanisms and results of optic–acoustic coupled biosensing technologies for coagulation testing. (**a**) Photoacoustic coagulation biosensing assay using water as medium. Figure reproduced from ref. [49]. CC BY license, Elsevier GmbH, copyright © 2023, the authors. (**b**) Design and mechanism of an in vivo acousto-optic biosensing method for fibrin monitoring based on all-optical ultrasound spectroscopic sensing: light and sound propagate through water, and the clot absorbs light and generates vibrations that are captured by acoustic sensors. Figure reproduced with permission from ref. [47]. Copyright © 2022, American Chemical Society.

Another representative approach is all-optical ultrasound-based spectral sensing (AOUSS). This technology uses laser irradiation of a dedicated transducer layer to excite broadband ultrasound, which propagates through the blood sample (Figure 6b) [47]. During coagulation, the formation of a fibrin network and adhesion and aggregation of cells alter the acoustic properties of the local medium, thereby modulating the ultrasound signal. The system employs a side-polished fiber sensor to detect the returned modulated ultrasound signal. From this signal, the system infers the blood viscoelastic state by analyzing changes in its spectral characteristics. Biswas et al. applied AOUSS to rat whole-blood samples and achieved highly sensitive detection of weak viscoelastic changes through spectral characteristics in the early clot stage. Meanwhile, the spectral parameters obtained by AOUSS exhibit a strong correlation with the viscoelastic metrics measured by rheometers. The AOUSS method translates mechanical/structural changes into acoustic spectral changes while preserving the advantages of optical detection of contactless and flexibility.

In contrast to the above hybrid platforms employing acoustic excitation and optical detection, photoacoustic (PA)-based coagulation biosensing technology adopts optical excitation and acoustic detection. PA directly utilizes the absorption of a short-pulse laser by strongly absorbing components such as RBCs and fibrin-rich thrombi or clots. Under the irradiation of a short-pulse laser at a specific wavelength, instantaneous thermoelastic expansion is induced in the strongly absorbing components, generating ultrasound signals with significant amplitude. Detection of the generated signals via an ultrasound transducer and spatial reconstruction enables high-contrast imaging of thrombi with a penetration depth of millimeters to centimeters. The miniaturized high-frequency PA biosensing system developed by Das et al. achieved blood flow imaging at a frame rate of kilohertz in vivo. This system enables differentiation of flowing blood from thrombi attached to blood vessel walls or device surfaces under high blood flow velocities [48]. In the in vitro mode, this PA biosensing system only requires only 1–25 μL of whole blood for a single detection (Figure 6a) [49]. To further reduce hardware costs and explore the miniaturization potential of PA systems for coagulation monitoring, Paul et al. adopted a detection scheme with a 2.25 MHz low-frequency transducer [50]. This system, from an engineering perspective, achieves a simplified acoustic front end and an engineering compromise between spatial resolution and penetration depth via multi-wavelength excitation.

In summary, optic–acoustic hybrid biosensing technology provides complementary capabilities to the aforementioned methods for coagulation monitoring. It can output interpretable viscoelastic curves and coagulation time readings in microvolume samples. It can also achieve spatial detection and identification of thrombi/microclots with enhanced penetration and absorption contrast, providing a novel imaging tool for deep thrombus assessment and interventional guidance. However, optic–acoustic hybrid biosensing technology is still in the early stage of transition from laboratory prototypes to clinical applications for coagulation detection. Like LS technologies, optic–acoustic hybrid technologies need more studies with multi-center, large-scale cohorts and more complex samples. Furthermore, there is no unified calibration and control system among metrics proposed by different platforms such as “reaction time”, “spectral sensitivity”, and “vibration parameters”; a stable conversion relationship with clinical reference standards such as CCT, TEG/ROTEM, and PT/INR has not been established. In addition, the physical implementation of these technologies usually requires precise control of sound and light fields and high system complexity, and it is sensitive to interference such as temperature changes, acoustic coupling status, bubbles, and environmental vibrations. These factors hinder their popularization and application in complex environments.

## 7. Fluorescence-Based Methods

Optical routes like texture, speckle, and OCT/OCE mainly rely on optical scattering or coherence signals to characterize structural and mechanical evolution. Different from that, fluorescence biosensing translates key molecular events within the coagulation cascade into quantifiable optical signals through engineerable fluorescent probe. This route enables direct reading of the activity of a single coagulation factor, drug target effects, and platelet function markers, and serves as an indispensable molecular monitoring method. Accordingly, fluorescence-based methods are not only suitable for mechanism research and pathway abnormality interpretation but also provide important tools for anticoagulant drug monitoring, personalized medication evaluation, and multi-index combined diagnosis.

From the perspective of sensing mechanisms, fluorescence-based detection related to coagulation can be roughly divided into three types of paths [1,51,52]. The first are enzyme-cleavable substrate probes. These probes, fluorophores or FRET pairs, are conjugated to specific substrates. Cleavage of substrate by target coagulation factors like thrombin, FXa, FXIa, and FXIIIa induces fluorescence enhancement or spectral characteristic changes. Measurements of signal intensities and kinetic parameters from these changes enable characterization of factor activity and even the inhibitory effect of DOACs on target enzyme activity. The second pathway is based on specific binding probes. These probes are antibodies, aptamers, or peptide probes that bind highly specifically to coagulation products/structures like fibrin, activated platelet epitopes, etc. This approach enables localization, quantification, and component interpretation through the spatial position, intensity, or ratio of fluorescence. The third is microenvironment-/morphology-responsive fluorescence reading. Gelation, network densification, and contraction during coagulation alter local viscosity, diffusion constraints, and spatial distribution. Leveraging environment-sensitive fluorescent dyes or spatiotemporal imaging of labeled components, the structure and deformation process can be converted into quantifiable kinetic indicators.

In clinically relevant applications, thrombin generation (TG) detection employs an enzyme-cleavable, specifically thrombin, fluorescence platform. TG involves real-time monitoring of the cleavage fluorescence under certain activation conditions. From the monitoring, key parameters, including lag time, peak thrombin, time to peak, and endogenous thrombin potential, are derived and are used to characterize overall coagulation potential and drug intervention effects [53]. In recent years, TG fluorescence platforms have been developed and have become compatible in whole-blood systems [54]. This facilitates the translation of TG by reducing manual operations, shortening processes, and reducing the impact of non-physiological factors such as contact activation.

**Figure 7:**
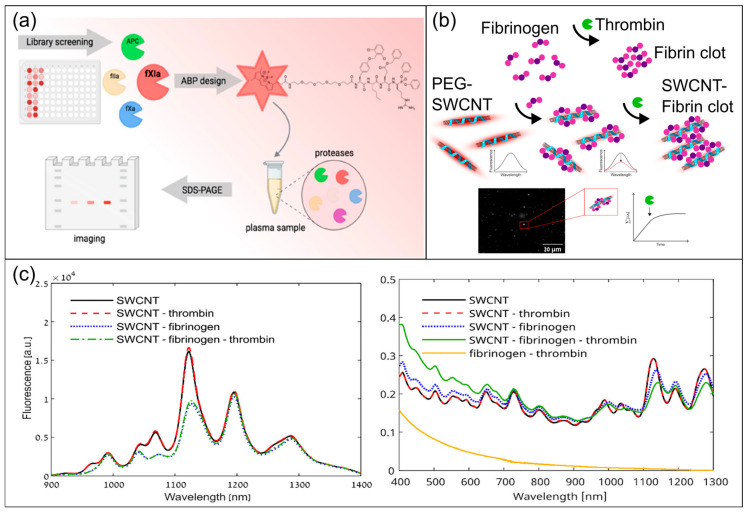
Example of recent advances in optical biosensors for coagulation monitoring based on fluorescence. (**a**) Novel design process of probes targeting factor XIa using peptide substrate screening and the incorporation of non-canonical amino acids. Figure reproduced from ref. [51]. CC BY 4.0 license, ACS Publications, copyright © 2023, the authors. (**b**) Working mechanism of fluorescence probes incorporating Single-Walled Carbon Nanotubes (SWCNTs). Figure reproduced from ref. [56]. CC BY 4.0 license, ACS Publications, copyright © 2023, the authors. (**c**) Fluorescence and absorption spectra upon thrombin and fibrinogen addition to SWCNT-based probes, indicating that fibrinogen remains bounded to SWCNT surfaces after thrombin addition, and its cleavage to fibrin plus subsequent polymerization does not alter the fluorescence of SWCNTs [56]. Figure reproduced from ref. [56]. CC BY 4.0 license, ACS Publications, copyright © 2023, the authors.

In response to the demand of “factor specificity and drug monitoring”, advances in probe engineering have significantly improved the resolution of fluorescence-based methods. For example, combinatorial peptide substrate screening and the incorporation of non-canonical amino acids have yielded fluorescent substrates/activity probes with high selectivity for target factor XIa (FXIa), thereby enhancing specificity in complex blood matrices (Figure 7a) [51]. Fluorescent probes for DOACs, specifically rivaroxaban, have enabled rapid quantitative or semi-quantitative evaluation under low-sample-volume conditions [55]. This probe engineering partially mitigates the sensitivity constraints of traditional chromogenic assays in the low-concentration range. Specific fluorescent peptide probes for FXIIIa have demonstrated the potential of reducing detection costs by reducing dependence on costly labeled substrates [52], going further preparing for future large-scale applications.

Beyond solution-phase factor activity reading, specific-binding- and imaging-based fluorescence strategies provide coagulation monitoring with spatial resolution. Structural components of clots, represented by fibrin, can be visualized through fluorescent probes in the near-infrared window. This enables imaging analysis of clot spatial distribution, formation kinetics, and even structural characteristics. The luminescent properties of nanomaterials like Single-Walled Carbon Nanotubes (SWCNTs) enhance in-tissue imaging and signal penetration, as these nanomaterials emit near-infrared fluorescence (Figure 7b,c) [56]. At the cellular level, the combination of specific fluorescence labeling and flow cytometry provides single-cell resolution in platelet function evaluation. By quantifying platelet surface receptor expression, activation markers, and granule release, this approach achieves highly sensitive detection in whole-blood matrices and even samples with low platelet counts [57]. Thereby, this approach helps to distinguish between hereditary and acquired platelet function defects, as well as drug-related effects.

Fluorescence methods can also be used for functional characterization of the terminal stage of clotting. For instance, clot retraction is a surrogate indicator of platelet contraction and clot mechanical stability. Clot retraction was quantified by tracking spatiotemporal dynamics of fluorescently labeled components, yielding parameters including retraction area, retraction rate, and final retraction degree [58]. These readouts are valuable in mechanism research and supplementary phenotypic indicators and further enable the deduction of correlations of molecules, structure, and function. Compatibility of this method with standard microplate reader processes improves assay throughput.

Fluorescence-based coagulation biosensing has remarkably developed in probe design, detection systems, and platform development, while its translation to routine clinical applications remains challenging. Firstly, cost and accessibility remain bottlenecks: custom synthesis of high-quality probes, specialized equipment such as flow cytometry/imaging, and quality control systems increase the total cost. Secondly, the coagulation network is highly complicated, with individual probes usually covering only segments of pathways. Achieving multi-factor and multi-pathway readouts while maintaining interpretability requires further methodological innovation. Thirdly, similar to LS, processes, thresholds, and calibration systems of fluorescence-based coagulation biosensing are not unified. Finally, like other early-stage optical technologies, most studies require larger and more complex cohorts as well as robustness testing and validation. Future directions can focus on low-cost and scalable probes, transferable multi-index calibration frameworks, and integration with microfluidics and automated analysis. These improvements could promote the evolution of fluorescence-based coagulation biosensing from mechanistic research tools to deployable clinical detection modules.

## 8. Emerging and Complementary Optical Methods

A series of emerging optical strategies based on unique physical mechanisms have also shown complementary value in coagulation detection. Rather than aiming to cover the entire coagulation process with a single platform, these methods address information that is difficult to directly obtain using mainstream technologies. These methods offer label-free molecular recognition, chemical composition fingerprinting, high-throughput phenotypic analysis of single cells, and robust monitoring under strong interference scenarios. Hence, these methods expand the observational scope and interpretative capacity of coagulation monitoring.

**Figure 8:**
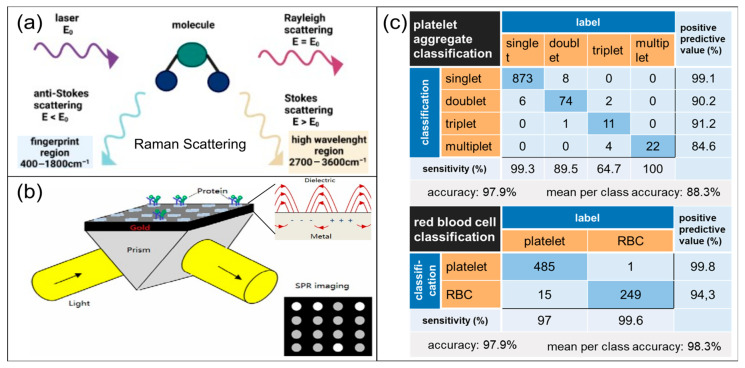
Example of other emerging optical biosensors for coagulation monitoring. (**a**) Schematic illustration of Raman scattering, core principle of Surface-Enhanced Raman Scattering. Figure reproduced from ref. [59]. CC BY license, MDPI Open Access, copyright © 2024. (**b**) Schematic of a surface plasmon resonance (SPR) imaging setup, depicting protein immobilization on a gold substrate and SPR signal detection. Figure reproduced with permission from ref. [64]. Copyright © 2012, Wiley-VCH Verlag GmbH & Co. KGaA Weinheim. (**c**) Confusion matrices for classification performance of red blood cell/platelet classification using convolutional neuronal network and flow cytometry. Figure reproduced from ref. [65]. CC BY NC license, Wiley Periodicals LLC, copyright © 2024, the authors. Cytometry Part A published by Wiley Periodicals LLC on behalf of International Society for Advancement of Cytometry.

In terms of label-free molecular recognition and component analysis, Raman spectroscopy uses inelastic scattering to provide molecular vibration, or chemical fingerprints [59,60], which can be used to characterize changes in coagulation-related components (Figure 8a) [61]. Surface-Enhanced Raman Scattering (SERS), using the local electromagnetic field enhancement of noble metal nanostructures, significantly improves the detection sensitivity of targets like thrombin and fibrinogen [62,63]. However, Raman-based methods are often constrained by weak signals, background interference, and matrix complexity in blood systems. This renders Raman-based methods more suited as supplementary tools for component typing, mechanism verification, and thrombus component research. They provide complementary chemical and compositional insights that explain or verify the sources of kinetic and mechanical changes reflected by methods like viscoelastic curves, speckle kinetics, and tomographic structures.

Classical techniques, such as surface plasmon resonance (SPR) and its localized form (LSPR), measure refractive index changes at the sensing interface to characterize molecular binding events (Figure 8b). These techniques thus enable real-time monitoring of coagulation-related protein binding processes [66]. Furthermore, integrated photonics platforms, such as silicon-based microring resonators, waveguide resonance structures, etc., can directly couple resonance wavelength shift and interface binding amount. These integrated platforms inherently enable array integration and multi-target parallel detection. This facilitates the translation of molecular layer affinity reactions into scalable chip-level detection modules [64,67]. Recent studies have attempted to detect key coagulation molecules of fibrinogen [68] and thrombin [69] using molecularly imprinted SPR and grating-coupled SPR respectively. Relative to the fluorescent probe route, these platforms provide advantages including label-free, quantifiable kinetics, and facile multiplexing. The critical prerequisites for their translation lie in the anti-fouling surface engineering under complex blood samples, correction to temperature drift and non-specific adsorption, and transferable calibration and threshold systems aligned with clinical gold standards. Complementary to the above resonance-based platforms is liquid crystal (LC) aptamer sensing. This technique achieves high-sensitivity detection with low instrument requirements. The causative mechanism is that LC orientation shifts rapidly when thrombin binds to its specific aptamer on LC, and amplified optical texture alterations can be observed [70]. These highlight the high sensitivity of interface amplification strategies, their material stability, and their potential in POC testing.

Meanwhile, AI-driven single-cell optical and optofluidic platforms are advancing platelet function analysis from overall curves to automated recognition of cell and aggregate phenotypes. Imaging flow cytometry or high-speed optofluidic imaging are combined with ML models such as CNNs, reducing confusion of traditional scattering readings in whole-blood environments. Such integration automatically identifies and quantifies platelet aggregates, activation-related morphological features, or cell complexes in complex backgrounds (Figure 8c) [65,71]. A key bottleneck of these approaches is usually the standardization of data distribution differences, annotation systems, and clinical thresholds across instruments or institutions.

Finally, in high-interference scenarios such as cardiopulmonary bypass and ECMO, multimodal anti-interference strategies have demonstrated clear engineering value. For example, magnetic flux is insensitive to oxygenation changes. Integration of magnetic flux and optical readouts allows for mutual verification and enhances the robustness of coagulation trend monitoring under variable oxygenation conditions [72]. These methods are primarily positioned for stable continuous monitoring in specific clinical scenarios. They emphasize anti-interference and usability and are thus suitable as engineering compensation and risk mitigation tools for mainstream optical signals in intraoperative or ICU environments.

In general, these emerging and complementary optical strategies collectively expand the observational dimensions of coagulation monitoring. Raman SERS emphasizes chemical composition and molecular fingerprints; SPR, LSPR, micro-rings and related integrated photonic platforms prioritize interfacial affinity and molecular kinetics; liquid crystal and interference methods emphasize amplification and anisotropy information; Artificial Intelligence (AI) single-cell platforms emphasize cell phenotype stratification; and multimodal systems emphasize robust deployment in complex scenarios. Notably, their common challenges towards clinical application are relatively consistent. Further refinements are required in mass-manufacturing reproducibility and system stability (especially for nano-substrates and interface engineering), background correction and interference mitigation strategies under complex matrices, and transferable control and threshold systems with gold standards. A more feasible advancement path involves explicitly positioning them as key supplementary modules for segmented tasks like ultra-trace target detection, drug response evaluation, thrombus component research, continuous monitoring of cardiopulmonary bypass. Gradually, an interpretable, comparable, and regulatory evidence chain could be established through standardization, quality control, and multi-center prospective validation.

## 9. Summary and Perspective

Hemostasis is not a binary switch of a single pathway but a multi-level dynamic equilibrium process involving procoagulation, anticoagulation, and fibrinolysis: molecular cascades determine pathway initiation and inhibition, platelets and cellular components shape early hemostatic plugs and reaction platforms, the formation and remodeling of fibrin networks define clot structure and stability, and the temporal evolution of clot viscoelasticity and fibrinolysis determine the final success of hemostasis and thrombosis risk [3]. Centered on this paradigm, the core contribution of optical coagulation biosensing is to decompose the “dynamic equilibrium” into observable, quantifiable, and mutually verifiable molecular–cellular–structural–mechanical/kinetic evidence chains. Herein, optical coagulation biosensing converts mechanism information into actionable clinical judgments through complementary integration.

Under this framework, different optical platforms exhibit distinct values and boundaries (summarized and compared in Table 1). LTA and imaging/texture are closer to rapid phenotyping at the cellular level and macro-process screening. LS and related correlation analyses are highly sensitive to microkinetic changes in the initial stage of coagulation and are thereby suitable for minute-level trend monitoring during the window at the start of the balance shift. OCT/OCE and optic-acoustic/PA platforms are more advantageous in structural and spatial positioning; hence, they reveal the three-dimensional heterogeneity of clot formation, boundary interactions, and local mechanical evolution. Fluorescence and affinity sensing provide irreplaceable specificity for molecular targets, drug effects, and pathway activities. Thus, coagulation detection is evolving from single-endpoint or single-curve readouts to a cross-scale, process-oriented, and interpretable characterization paradigm. However, it must be acknowledged that different platforms have inevitable trade-offs between information depth, timeliness, cost, and deployability. A more feasible pathway is likely to be modularly complementary rather than an all-in-one platform.

The translation of optical coagulation sensing from research to clinical systems is still constrained by three key barriers. Firstly, a crucial barrier is an insufficient evidence chain. Most studies remain limited to single-center, small-sample and comparatively homogenous populations. They have insufficient systematic validation covering populations with complex traumatic coagulopathy, rare coagulation disorders, perioperative multi-factor interventions, and specific medication populations such as DOACs. Furthermore, prospective evidence associated with clinical outcomes, like bleeding, thrombosis events, blood transfusion volume, and ICU stay time, remains scarce. Secondly, another significant barrier lies in the lack of endpoints and standardization in research and applications. To evaluate hemostatic dynamic equilibrium, optical metrics must be anchored to clinically universal language and decision nodes. Specifically, these metrics include decorrelation time, texture threshold, phase stability, wave speed/modulus, fluorescence kinetics, etc. The optical metrics require unified metrologically traceable frameworks, quality control specifications, cross-device comparability frameworks, transferable mapping models, and thresholds associated with established standards like PT/aPTT (INR), ACT, TEG/ROTEM, etc. Thirdly, translation requires robustness and interference correction. Temperature drift, vibration, and changes in coupling status in real clinical environments, as well as sample interference like HIL, fluctuations in hematocrit at the sample level, etc., can introduce systematic deviations in high-sensitivity optical readings. Without real-time quality control and correction mechanisms, the judgment of dynamic equilibrium may instead be driven by interference and deviations.

Therefore, the key to advancing the clinical implementation of optical coagulation biosensing is to form an engineerable integration strategy around the goal of continuous evaluation of dynamic equilibrium. At the engineering level, a robustness framework of “passive protection plus active compensation” could be established [73]. On one hand, environmental drift should be mitigated through structure and optical path solidification, temperature control, and anti-vibration design. On the other hand, matrix interference should be suppressed through physical strategies such as reference channels, ratio reading, dual-wavelength/spectral correction, and traceable quality control process. Along this core paradigm, the role of AI and data science should also evolve from fitting results to process understanding [74,75,76]. AI and data science could be used not only for improving accuracy but also for interference identification, domain adaptation, prediction based on early output, and multimodal fusion. This could empower the system to give trend judgments or risk prompts when the coagulation curve is not fully formed while maintaining interpretability and generalizability. Additionally, the value of integration with microfluidics and automation extends beyond miniaturization. Rather, microfluidics and automation standardize sample processing, activation conditions, and shear environments, reduce contact activation and human differences, and realize a closed process of “sample in, result out”. Thereby microfluidics and automation improve the deployablity of the continuous monitoring of the dynamic equilibrium for POC applications.

Looking forward, the ultimate goal of coagulation monitoring should not be the replacement of traditional systems by a single technology but the establishment of a cross-level “structure–function–molecule” closed loop around dynamic equilibrium, capturing early kinetic shifts with LS, providing structural and local mechanical positioning with OCT/OCE/optic–acoustic platforms, characterizing molecular pathways and drug responses with fluorescence/affinity modules, and realizing mutual verification and integration under a unified calibration and quality control framework, ultimately outputting actionable indicators directly related to treatment adjustments. The advancement pathway can be summarized as three steps. In the short term, endpoint definition and gold standard mapping should be completed through controlled studies, and interference identification and quality control specifications should be established. In the medium term, modular integration and multi-center external validation should be realized around high-value scenarios like trauma/intraoperative, cardiopulmonary bypass/ECMO, and anticoagulant management. In the long term, closed-loop decision support based on multimodal fusion and real-time prediction should be formed, and its net benefit on blood transfusion strategies, anticoagulant adjustments, and bleeding/thrombosis outcomes should be proven through prospective outcome studies. With the improvement of standardization systems, maturation of engineering and miniaturization, and accumulation of high-quality multi-center evidence, optical coagulation biosensing is expected to truly bring dynamic equilibrium from a concept into a measurable, interpretable, and intervenable clinical tool, unlocking its unique value in personalized hemostatic and antithrombotic management.

## Figures and Tables

**Table 1:** Comparison of key performance parameters among coagulation detection methods.

Method	Detection Scope	Anti-Environment Interference	Anti-Sample Interference	Lowest Sample Volume	Test Time	Sensitivity	Cost of Test and Apparatus
TEG/ROTEM	Whole-blood global coagulation by measuring change in mechanical resistance	moderate	high	traditional: 340–360 μL traditional	~15 min, traditional: 30–60 min	moderate	moderate
Stepwise Laboratory Screening	Plasma-based pathway-specific endpoints	low	medium	100~ μL PRP	1–5 h	high	relatively high
LTA	Platelet aggregation in platelet-rich plasma	low	low	50–100 μL PRP	~10 min	moderate, platelet function only	low
Fluorescence	Molecular-level coagulation factor activity; fibrin generation	low	low	10–50 μL	1–10 min	high	high
OCT/OCE	Clot microstructure; depth-resolved structural dynamics; OCE quantifies mechanical resistance	low	low	100–500 μL	5–20 min	moderate	relatively high
LS (Laser Speckle)	Whole-blood rheological dynamics and viscosity measurement; speckle decorrelation reflects scatterer motion	low	low	50–100 μL	0.5–10 min	high	low
Texture Detection	Whole-blood macroscopic coagulation measuring texture change or using image processing	low	low	50–200 μL	25 s–30 min	low	low
Acoustic–Optical Hybrid	Whole-blood viscoelasticity; deep-tissue microclot detection with photoacoustic effect of clot	low	low	1–25 μL, in vivo	3–15 min	high	high

## Data Availability

All data discussed are derived from published studies cited in the references, which are accessible via standard academic databases.

## Abbreviations

The following abbreviations are used in this manuscript:

ECMO	Extracorporeal Membrane Oxygenation
VET	Viscoelastic Testing
PT	Prothrombin Time
aPTT	activated Partial Thromboplastin Time
INR	International Normalized Ratio
FIB	Fibrinogen
TT	Thrombin Time
ACT	Activated Clotting Time
TEG	Thromboelastography
ROTEM	Rotational Thromboelastometry
MA	Maximum Amplitude
LTA	Light Transmission Aggregometry
ML	Machine Learning
LS	Laser Speckle
LSR	Laser Speckle Rheology
OCT	Optical Coherence Tomography
OCE	Optical Coherence Elastography
PRP	Platelet-Rich Plasma
PPP	Platelet-Poor Plasma
POC	Point-Of-Care
RBC	Red Blood Cell
CNN	Convolutional Neural Network
HIL	Hemolysis, Icterus, Lipemia
CCT	Conventional Coagulation Test
ICU	Intensive Care Unit
DOAC	Direct Oral Anticoagulant
AOUSS	All-Optical Ultrasound-based Spectral Sensing
PA	Photoacoustic
TG	Thrombin Generation
SPR	Surface Plasmon Resonance
LSPR	Localized Surface Plasmon Resonance
AI	Artificial Intelligence

## References

[1] Mohammadi Aria M., Erten A., Yalcin O. (2019). Technology Advancements in Blood Coagulation Measurements for Point-of-Care Diagnostic Testing. Front. Bioeng. Biotechnol..

[2] Nadkarni S.K. (2019). Comprehensive Coagulation Profiling at the Point-of-Care Using a Novel Laser-Based Approach. Semin. Thromb. Hemost..

[3] Yong J., Toh C.H. (2023). Rethinking coagulation: From enzymatic cascade and cell-based reactions to a convergent model involving innate immune activation. Blood.

[4] Perez-Calatayud A.A., Hofmann A., Pérez-Ferrer A., Escorza-Molina C., Torres-Pérez B., Zaccarias-Ezzat J.R., Sanchez-Cedillo A., Paez-Zayas V.M., Carrillo-Esper R., Görlinger K. (2023). Patient Blood Management in Liver Transplant-A Concise Review. Biomedicines.

[5] Thiele E.L., Forkin K.T. (2024). Update on Coagulation Monitoring in Liver Transplantation. Curr. Anesthesiol. Rep..

[6] Caspers M., Holle J.F., Limper U., Froehlich M., Bouillon B. (2022). Global Coagulation Testing in Acute Care Medicine: Back to Bedside?. Hamostaseologie.

[7] Siniarski A., Gasecka A., Borovac J.A., Papakonstantinou P.E., Bongiovanni D., Ehrlinder H., Giustozzi M., Guerreiro R.A., Parker W.A.E. (2023). Blood Coagulation Disorders in Heart Failure: From Basic Science to Clinical Perspectives. J. Card. Fail..

[8] Lester W., Bent C., Alikhan R., Roberts L., Gordon-Walker T., Trenfield S., White R., Forde C., Arachchillage D.J. (2024). A British Society for Haematology guideline on the assessment and management of bleeding risk prior to invasive procedures. Br. J. Haematol..

[9] Riley R.S., Rowe D., Fisher L.M. (2000). Clinical utilization of the international normalized ratio (INR). J. Clin. Lab. Anal..

[10] Hartmann J., Murphy M., Dias J.D. (2020). Viscoelastic Hemostatic Assays: Moving from the Laboratory to the Site of Care-A Review of Established and Emerging Technologies. Diagnostics.

[11] Gorio C., Molinari A.C., Martini T., Ferretti A., Albrici G., Carracchia G., Ierardi A., Leotta M., Portesi N., Sacco M. (2025). Hemostasis Laboratory Diagnostics in Newborns. J. Clin. Med..

[12] Dias J.D., Levy J.H., Tanaka K.A., Zacharowski K., Hartmann J. (2025). Viscoelastic haemostatic assays to guide therapy in elective surgery: An updated systematic review and meta-analysis. Anaesthesia.

[13] Coggins A.R., Nguyen V.D.D., Pasalic L., Ramesh M., Wangoo K. (2025). Utility of point of care viscoelastic haemostatic assays for trauma patients in the emergency department. Scand. J. Trauma Resusc. Emerg. Med..

[14] Volod O., Bunch C.M., Zackariya N., Moore E.E., Moore H.B., Kwaan H.C., Neal M.D., Al-Fadhl M.D., Patel S.S., Wiarda G. (2022). Viscoelastic Hemostatic Assays: A Primer on Legacy and New Generation Devices. J. Clin. Med..

[15] Gebetsberger J., Prüller F. (2024). Classic Light Transmission Platelet Aggregometry: Do We Still Need it?. Hamostaseologie.

[16] Koltai K., Kesmarky G., Feher G., Tibold A., Toth K. (2017). Platelet Aggregometry Testing: Molecular Mechanisms, Techniques and Clinical Implications. Int. J. Mol. Sci..

[17] McGlasson D.L., Fritsma G.A. (2009). Whole blood platelet aggregometry and platelet function testing. Semin. Thromb. Hemost..

[18] Le Blanc J., Mullier F., Vayne C., Lordkipanidzé M. (2020). Advances in Platelet Function Testing—Light Transmission Aggregometry and Beyond. J. Clin. Med..

[19] Hsu H., Chan M., Armstrong P.C., Crescente M., Donikian D., Kondo M., Brighton T., Chen V., Chen Q., Connor D. (2022). A pilot study assessing the implementation of 96-well plate-based aggregometry (Optimul) in Australia. Pathology.

[20] Kim C.-J., Kim J., Sabaté del Río J., Ki D.Y., Kim J., Cho Y.-K. (2021). Fully automated light transmission aggregometry on a disc for platelet function tests. Lab A Chip.

[21] Sharma P., Sachdeva M.U.S., Kumar N., Bose S., Bose P., Uppal V., Malhotra P., Bansal D., Varma N., Ahluwalia J. (2021). A comparative study between light transmission aggregometry and flow cytometric platelet aggregation test for the identification of platelet function defects in patients with bleeding. Blood Res..

[22] Xu O., Hartmann J., Tang Y.D., Dias J. (2022). The Use of Thromboelastography in Percutaneous Coronary Intervention and Acute Coronary Syndrome in East Asia: A Systematic Literature Review. J. Clin. Med..

[23] Louka M., Kaliviotis E. (2021). Development of an Optical Method for the Evaluation of Whole Blood Coagulation. Biosensors.

[24] Xu W., Althumayri M., Mohammad A., Ceylan Koydemir H. (2023). Foldable low-cost point-of-care device for testing blood coagulation using smartphones. Biosens. Bioelectron..

[25] Chen L., Yu L., Liu Y., Xu H., Ma L., Tian P., Zhu J., Wang F., Yi K., Xiao H. (2022). Space-time-regulated imaging analyzer for smart coagulation diagnosis. Cell Rep. Med..

[26] Lapić I., Lončar Vrančić A., Coen Herak D., Rogić D. (2021). The missing slope: Paradoxical shortening of activated partial thromboplastin time in a patient on unfractionated heparin therapy. Biochem. Medica.

[27] Gong J., Zhang Y., Zhang H., Li Q., Ren G., Lu W., Wang J. (2022). Evaluation of Blood Coagulation by Optical Vortex Tracking. Sensors.

[28] Han J.H., Yoon I., Jeon H.J. (2024). Microfluidic System-Based Quantitative Analysis of Platelet Function through Speckle Size Measurement. Biomolecules.

[29] Tripathi M.M., Hajjarian Z., Van Cott E.M., Nadkarni S.K. (2014). Assessing blood coagulation status with laser speckle rheology. Biomed. Opt. Express.

[30] Tripathi M.M., Tshikudi D.M., Hajjarian Z., Hack D.C., Van Cott E.M., Nadkarni S.K. (2020). Comprehensive Blood Coagulation Profiling in Patients Using iCoagLab: Comparison Against Thromboelastography. Thromb. Haemost..

[31] Hoare D., Zeng Z., Foster E., Hai N., Nadkarni S. iCoagLAB permits comprehensive coagulation profiling in patients with percutaneous microaxial pump support. Proceedings of the Volume PC13295, Diagnostic and Therapeutic Applications of Light in Cardiology 2025.

[32] Patiño-Velasco M.M., Andrade-Eraso C., Vásquez-López J., Trivi M., Rabal H.J. (2015). Blood Coagulation Measurements Using Dynamic Speckle Technique. VI Latin American Congress on Biomedical Engineering CLAIB 2014, Paraná, Argentina, 29–31 October 2014.

[33] Li L., Sytnik I.D., Gubarev F.A., Pekker Y.S. (2018). Evaluation of Blood Plasma Coagulability by Laser Speckle Correlation. Biomed. Eng..

[34] Liushnevskaya Y.D., Gubarev F.A., Li L., Nosarev A.V., Gusakova V.S. (2020). Measurement of Whole Blood Coagulation Time by Laser Speckle Pattern Correlation. Biomed. Eng..

[35] Guzman-Sepulveda J.R., Argueta-Morales R., DeCampli W.M., Dogariu A. (2017). Real-time intraoperative monitoring of blood coagulability via coherence-gated light scattering. Nat. Biomed. Eng..

[36] Batarseh M., Guzman-Sepulveda J.R., Wu R., DeCampli W.M., Dogariu A. (2020). Passive Coagulability Assay Based on Coherence-Gated Light Scattering. Hemato.

[37] Guzman-Sepulveda J.R., DeCampli W.M., Dogariu A. Intraoperative Assessment of Blood Coagulability using Coherence-gated Light Scattering. Proceedings of the Biophotonics Congress: Biomedical Optics Congress 2018 (Microscopy/Translational/Brain/OTS).

[38] Bouma B.E., de Boer J.F., Huang D., Jang I.-K., Yonetsu T., Leggett C.L., Leitgeb R., Sampson D.D., Suter M., Vakoc B.J. (2022). Optical coherence tomography. Nat. Rev. Methods Primers.

[39] Tang Y., Ma Z., Zhang Y., Fan F., Zhang F., Zhu J. (2025). Noncontact Detection of Blood Coagulation Dynamics Based on Speckle Deviation Analysis Using Optical Coherence Tomography. J. Biophotonics.

[40] Tang Y., Zhu J., Zhu L., Fan F., Ma Z., Zhang F. (2022). Blood coagulation monitoring under static and flow conditions with optical coherence tomography autocorrelation analysis. Appl. Phys. Lett..

[41] Li Y., Li W., Zhang X., Lin H., Li D., Li Z. (2024). Three-dimensional morphological characterization of blood droplets during the dynamic coagulation process. J. Biophotonics.

[42] Xu X., Zhu J., Chen Z. (2016). Dynamic and quantitative assessment of blood coagulation using optical coherence elastography. Sci. Rep..

[43] Singh M., Hepburn M.S., Kennedy B.F., Larin K.V. (2025). Optical coherence elastography. Nat. Rev. Methods Primers.

[44] Xu X., Zhu J., Yu J., Chen Z. (2019). Viscosity monitoring during hemodiluted blood coagulation using optical coherence elastography. IEEE J. Sel. Top. Quantum Electron. A Publ. IEEE Lasers Electro-Opt. Soc..

[45] Cuartas-Vélez C., Middelkamp H.H.T., van der Meer A.D., van den Berg A., Bosschaart N. (2023). Tracking the dynamics of thrombus formation in a blood vessel-on-chip with visible-light optical coherence tomography. Biomed. Opt. Express.

[46] Kasireddy N., Luo D., Khismatullin D.B. (2024). Whole blood PT/aPTT assay based on non-contact drop-of-sample acoustic tweezing spectroscopy. Anal. Bioanal. Chem..

[47] Biswas D., Heo J., Sang P., Dey P., Han K., Ko J.H., Won S.M., Son D., Suh M., Kim H.S. (2022). Micro-ultrasonic Assessment of Early Stage Clot Formation and Whole Blood Coagulation Using an All-Optical Ultrasound Transducer and Adaptive Signal Processing Algorithm. ACS Sens..

[48] Das D., Sivasubramanian K., Rajendran P., Pramanik M. (2021). Label-free high frame rate imaging of circulating blood clots using a dual modal ultrasound and photoacoustic system. J. Biophotonics.

[49] Bodera F.J., McVey M.J., Sathiyamoorthy K., Kolios M.C. (2023). Detection of clot formation & lysis In-Vitro using high frequency photoacoustic imaging & frequency analysis. Photoacoustics.

[50] Paul S., Khanam Z., Roy M., Patel H.S., Misra V., Rani R., Sahoo A.K., Saha R.K. An experimental investigation on in-vitro blood clot formation using multi-wavelength photoacoustics. Proceedings of the 2024 IEEE South Asian Ultrasonics Symposium (SAUS).

[51] Modrzycka S., Kołt S., Adams T.E., Potoczek S., Huntington J.A., Kasperkiewicz P., Drąg M. (2023). Fluorescent Activity-Based Probe To Image and Inhibit Factor XIa Activity in Human Plasma. J. Med. Chem..

[52] Liu Y., Crossen J., Stalker T.J., Diamond S.L. (2024). Fluorescent peptide for detecting factor XIIIa activity and fibrin in whole blood clots forming under flow. Res. Pract. Thromb. Haemost..

[53] Tripodi A. (2016). Thrombin Generation Assay and Its Application in the Clinical Laboratory. Clin. Chem..

[54] Wan J., Konings J., Yan Q., Kelchtermans H., Kremers R., de Laat B., Roest M. (2020). A novel assay for studying the involvement of blood cells in whole blood thrombin generation. J. Thromb. Haemost..

[55] Kim P.Y., Di Giuseppantonio L.R., Wu C., Douketis J.D., Gross P.L. (2019). An assay to measure levels of factor Xa inhibitors in blood and plasma. J. Thromb. Haemost..

[56] Gerstman E., Hendler-Neumark A., Wulf V., Bisker G. (2023). Monitoring the Formation of Fibrin Clots as Part of the Coagulation Cascade Using Fluorescent Single-Walled Carbon Nanotubes. ACS Appl. Mater. Interfaces.

[57] Spurgeon B.E.J., Linden M.D., Michelson A.D., Frelinger A.L. (2021). Immunophenotypic Analysis of Platelets by Flow Cytometry. Curr. Protoc..

[58] Peshkova A.D., Weisel J.W., Litvinov R.I. (2024). A novel technique to quantify the kinetics of blood clot contraction based on the expulsion of fluorescently labeled albumin into serum. J. Thromb. Haemost..

[59] Laskowska P., Mrowka P., Glodkowska-Mrowka E. (2024). Raman Spectroscopy as a Research and Diagnostic Tool in Clinical Hematology and Hematooncology. Int. J. Mol. Sci..

[60] Atkins C.G., Buckley K., Blades M.W., Turner R.F.B. (2017). Raman Spectroscopy of Blood and Blood Components. Appl. Spectrosc..

[61] Poon K.W.C., Lyng F.M., Knief P., Howe O., Meade A.D., Curtin J.F., Byrne H.J., Vaughan J. (2012). Quantitative reagent-free detection of fibrinogen levels in human blood plasma using Raman spectroscopy. Analyst.

[62] Hu J., Zheng P.C., Jiang J.H., Shen G.L., Yu R.Q., Liu G.K. (2009). Electrostatic interaction based approach to thrombin detection by surface-enhanced Raman spectroscopy. Anal. Chem..

[63] Wang X., Huang S.-C., Hu S., Yan S., Ren B. (2020). Fundamental understanding and applications of plasmon-enhanced Raman spectroscopy. Nat. Rev. Phys..

[64] Guo X. (2012). Surface plasmon resonance based biosensor technique: A review. J. Biophotonics.

[65] Poschkamp B., Bekeschus S. (2024). Convolutional neuronal network for identifying single-cell-platelet-platelet-aggregates in human whole blood using imaging flow cytometry. Cytometry Part A: J. Int. Soc. Anal. Cytol..

[66] Hojjat Jodaylami M., Masson J.-F., Badia A. (2025). Surface plasmon resonance sensing. Nat. Rev. Methods Primers.

[67] Homola J. (2003). Present and future of surface plasmon resonance biosensors. Anal. Bioanal. Chem..

[68] Zhu L., Gong Y., Wang Y. (2025). Poly(2-methyl-2-oxazoline)-grafted molecularly imprinted surface plasmon resonance biosensors enabling real-time fibrinogen detection. Eur. Polym. J..

[69] Kotlarek D., Vorobii M., Ogieglo W., Knoll W., Rodriguez-Emmenegger C., Dostálek J. (2019). Compact Grating-Coupled Biosensor for the Analysis of Thrombin. ACS Sens..

[70] Kim H., An Z., Jang C.-H. (2018). Label-free optical detection of thrombin using a liquid crystal-based aptasensor. Microchem. J..

[71] Jiang Y., Lei C., Yasumoto A., Kobayashi H., Aisaka Y., Ito T., Guo B., Nitta N., Kutsuna N., Ozeki Y. (2017). Label-free detection of aggregated platelets in blood by machine-learning-aided optofluidic time-stretch microscopy. Lab A Chip.

[72] Sakamoto H., Hibino N., Mizukuchi Y., Sato A., Torii T. (2024). Detection of blood coagulation in an extracorporeal circuit using magnetic and absorbance properties. AIP Adv..

[73] Lian X., Luo L., Dong M., Miao Z., Qi X., Cai Z., Wang L. (2024). A review on the recent progress on photodetectors. J. Mater. Sci..

[74] Guo J., Wu X., Liu J., Wei T., Yang X., Yang X., He B., Zhang W. (2021). Non-contact vibration sensor using deep learning and image processing. Measurement.

[75] Liao G., Caravaca-Mora O., Rosa B., Zanne P., Dall’Alba D., Fiorini P., de Mathelin M., Nageotte F., Gora M.J. (2022). Distortion and instability compensation with deep learning for rotational scanning endoscopic optical coherence tomography. Med. Image Anal..

[76] Pan Y., Luo Q., Fan Y., Chen H., Zhou D., Luo H., Jiang W., Su J. (2025). Deep Learning-Based Denoising of Noisy Vibration Signals from Wavefront Sensors Using BiL-DCAE. Sensors.

